# Unveiling the Subterranean Symphony: A Comprehensive Study of Cave Fungi Revealed Through National Center for Biotechnology Sequences

**DOI:** 10.3390/jof11040286

**Published:** 2025-04-05

**Authors:** Željko Savković, Slađana Popović, Miloš Stupar

**Affiliations:** Faculty of Biology, University of Belgrade, Studentski Trg 16, 11000 Belgrade, Serbia; zsavkovic@bio.bg.ac.rs (Ž.S.); sladjana.popovic@bio.bg.ac.rs (S.P.)

**Keywords:** caves, diversity, multivariate analyses, mycobiota, speleomycology

## Abstract

Caves can be regarded as extreme environments, and fungi are known as omnipresent and highly adaptable organisms that can easily colonize such environments. The primary objective of this study was to use the statistical analysis of sequences stored in the NCBI database, together with related metadata, to find and uncover statistically significant distribution patterns of fungi occupying different substrata inside the caves. The obtained list included a total of 1447 sequences corresponding to fungi isolated from various substrata within cave environments around the world, which corresponds to 445 fungal species, members of the 394 genera. Ascomycota was the most dominant phylum and Eurotiomycetes the dominant class of fungal dwellers in these environments. The highest species richness is detected for the genus *Penicillium* (57), followed by *Aspergillus* (51). On the other hand, the most frequently documented single species was *Pseudogymnoascus destructans*, isolated mostly from hibernating bats and guano, followed by *Penicillium chrysogenum*. Because caves have stable, nutrient-limited, low-competition microhabitats that support unusual or cryptic species, many new fungal taxa have been reported as well (such as *Aspergillus*, *Apiotrichum*, and *Cephalotrichum* species). Finally, cutting-edge molecular technologies and better sampling methods are revealing hitherto undiscovered fungal diversity in caves worldwide.

## 1. Introduction

Fungi can colonize any organic substrata within the caves; they can establish different biotic interactions with other cave organisms and are among the most dominant organisms [1]. Speleomycology is a novel scientific discipline that studies the diversity, ecology, and role of cave mycobiota [2]. The significance of cave fungi can therefore be summarized in the following points:

1. Saprobe fungi are the main decomposers of organic matter in subterranean sites with a significant role in the biogeochemical cycling of elements in caves [3]. Previous speleomycological studies have suggested that fungi can colonize a variety of organic substrata within the caves, such as bat guano, moonmilk, sediments, etc. Also, cave fungi can be isolated from carcasses of cave-associated animals, which are divided into troglobites (obligate cave dwellers), troglophiles (organisms that can live and reproduce in caves but also in surface habitats with similar conditions), and trogloxenes (organisms that use caves occasionally but do not depend on them for survival) [4].
2. Some fungal species are parasites and can cause diseases of cave animals. For example, *Pseudogymnoascus destructans* (Ascomycota, Leotiomycetes) is a causative agent of the white-nose syndrome in bats [5]. Likewise, the members of genus *Arthrorhynchus* (Ascomycota, Laboulbeniomycetes) are obligate parasites of bat flies and are documented in various caves [6].
3. Fungi can serve as a food source for various cave-dwelling organisms. In cave ecosystems with limited external inputs, fungi may represent a crucial energy and nutrient source for certain species, including cave-adapted invertebrates [1].
4. Fungi are constituents of subaerial biofilms (SAB), which are often formed on cave walls and ceilings. The metabolic activity of SAB-forming fungi could lead to weathering of stone substrata, unusual colorations on speleothems, as well as precipitation and formation of corrosion residues [7].
5. Many fungal species are the main “culprits” responsible for the biodeterioration of prehistoric wall paintings in the caves with Paleolithic art [8]. Also, in the caves that are repurposed for sacral objects during the history of mankind, fungi can be biodeteriogens of murals and other artifacts deposited within [9,10]. Nevertheless, biodeterioration induced by fungi is reported for several caves from the UNESCO list of world cultural heritage sites. For example, black stains, as a biodeterioration symptom of fungal origin, are reported on walls with prehistoric paintings in the Lascaux cave [8].
6. Fungal propagules, mostly spores and mycelial fragments, are always present in the cave air, and when optimal conditions are met, they could reach high concentrations expressed in CFU m^−3^ of air [11]. In fact, high concentrations of toxigenic and pathogenic airborne fungi in the caves are linked with impaired indoor air quality, and this issue could be regarded as a health risk factor for both visitors and cave personnel in show caves [12].
7. Caves could be referred to as extreme environments, poor in nutrients, which is a limiting factor for the growth and proliferation of fungi, which are heterotrophs. Although the number of speleomycological studies constantly increases, caves are still considered biologically poorly investigated environments and a potential source for novel fungal species. For instance, *Scolecobasidium lascauxense* (syn. *Ochroconis lascauxensis*) and *S. anomalum* (syn. *O. anomala*), members of Dothideomycetes, are melanized micromycetes that were first documented, isolated, and described from the above-mentioned black stains of Paleolithic paintings in the famous Lascaux cave [13]. Furthermore, Zhang et al. [14] described 20 novel fungal species in karst caves across China. Although some fungal species, such as *Acaulium caviariforme* (Ascomycota, Sordariomycetes), *Aspergillus baeticus*, and *A. thesauricus* (Ascomycota, Eurotiomycetes), are only documented in cave environments, it is still discussed whether truly there exist fungi that could be named obligate troglobionts [15].
8. It is a well-known fact that the presence of fungi in caves contributes to the overall biodiversity of subterranean ecosystems. Also, some fungal species reported in caves may have unique biochemical properties and be the potential source for novel organic compounds. In this sense, these fungi can be of interest for biotechnological applications, especially in the pharmaceutical industry, due to the production of metabolites with different biological activities [16].

There are few review papers regarding cave fungi. The pioneering work in this field is presented by Rutherford and Huang [17]. Furthermore, Vanderwolf et al. [18] presented the list of 1029 species of filamentous fungi, yeasts, and slime molds from a variety of substrata in caves and mines obtained after a comprehensive search of peer-reviewed literature. Kato et al. [19] highlighted multi-omics integration as a novel strategy to achieve new insights and perspectives about the biology, chemistry, and microbiology of caves. An interesting review article was presented by Cyske et al. [16]. These authors regarded the caves as “hard-to-reach environments” and, as such, a potential source of microorganisms, mostly bacteria, but also fungi, which are producers of various biologically active compounds. Also, Barbosa et al. [20] reviewed the biotechnological potential of cave fungi and reported the broad potential of compounds, antioxidants, antitumor agents, enzymes, and organic acids of cave-dwelling fungi. Additionally, Bontemps et al. [21] reviewed the microbial ecology of touristic caves with Paleolithic art, emphasizing high-throughput sequencing as a novel scientific approach in the study of cave microbiota.

To our knowledge, there are no review papers dealing with cave mycobiota that collect data from the National Center for Biotechnology (NCBI) database. The main goal of this paper is to search for and reveal statistically significant distribution patterns of fungi colonizing various substrata within the caves through the statistical analysis of sequences deposited in the NCBI database along with accompanying metadata.

## 2. Materials and Methods

### 2.1. NCBI Database Search

In order to extract nucleotide sequences from the NCBI database of all fungi related to the cave environment, advanced search options were applied on the following website: https://www.ncbi.nlm.nih.gov/ (accessed on 22 May 2023). A search query with relevant keywords separated with Boolean operators (AND and OR) took place in May 2023. To obtain sequences of fungal species present in the caves, taxonomic identifiers were used as target keywords—“Fungi” [Organism], along with relevant keywords and database fields. For the sequence type, “ITS” [All Fields] OR “rRNA” [All Fields] are used. Habitat-related terms such as “cave”, “subterranean”, “cavern”, and “guano” were used as additional keywords in the search query.

The NCBI database search was performed by all three review authors independently. The search output was extracted as a FASTA file from NCBI and sorted in an Excel file that contained accompanying metadata (accession number, isolation source, country of isolation, strain, host, etc.). Furthermore, additional searches in Web of Science (http://isiknowledge.com (accessed on 19 June 2023)), PubMed, Google Scholar, and Scopus were used to link NCBI outputs to corresponding peer-reviewed publications, while DOI and title of publication were added in the form (Supplementary Table S1, with references [1,12,14,22,23,24,25,26,27,28,29,30,31,32,33,34,35,36,37,38,39,40,41,42,43,44,45,46,47,48,49,50,51,52,53,54,55,56,57,58,59,60,61,62,63,64,65,66,67,68,69,70,71,72,73,74,75,76,77,78,79,80,81,82,83,84,85,86,87,88,89,90,91,92,93,94,95,96,97,98,99,100,101,102,103,104,105]). However, the unpublished data from NCBI were also used in this study only in the cases where the isolation_source in the NCBI database refers unambiguously to cave substrata (i.e., cave sediment, cave soil, cave wall, guano…) and if they provided sufficient additional information (i.e., geo_loc_name, host…) that could be used for statistical analyses.

### 2.2. Statistical Anlyses

Multivariate analyses were performed in the software Canoco 5 [106]. In these analyses, individual fungal representatives, fungal classes, and phyla were used as response data. The main data set contained the list of fungal representatives that are mentioned in the NCBI database. The option “trait averages” was used in the software to group individual taxa in the corresponding higher taxonomic units—classes and phyla. When using redundance analysis (RDA), fungal phyla, as well as fungal classes, were observed in relation to the continent and substratum separately. When considering individual taxa, only those with a number of more than ten isolates were observed in relation to the continent by using canonical correspondence analysis (CCA). The substrata categorized included the terms found in the paper and are presented in Supplementary File S1. The frequencies of taxa presented in pie diagrams were composed in Microsoft Office Excel 2021.

## 3. Results

**Figure 1:**
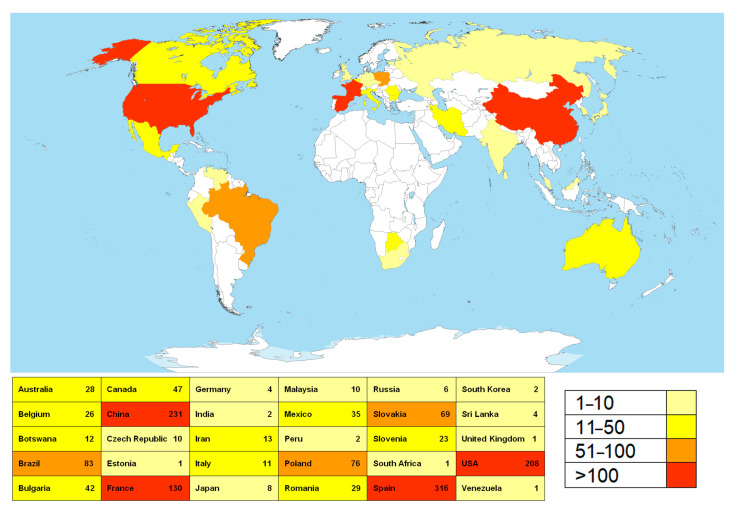
The countries with the highest number of sequences of cave fungi deposited to the NCBI database. (The map was downloaded and modified from the following: https://en.wikipedia.org/wiki/File:A_large_blank_world_map_with_oceans_marked_in_blue.PNG (accessed on 28 February 2025); permission is granted to copy, distribute, and/or modify this document under the terms of the GNU Free Documentation License, Version 1.2 or any later version published by the Free Software Foundation (https://commons.wikimedia.org/wiki/Commons:GNU_Free_Documentation_License,_version_1.2 (accessed on 28 February 2025)); this file is licensed under the Creative Commons Attribution-Share Alike 3.0 Unported license (https://creativecommons.org/licenses/by-sa/3.0/deed.en (accessed on 28 February 2025)).

A total of 22,407 sequences were retrieved from the NCBI base. After the filtration, the list was reduced to 1447 sequences corresponding to fungi isolated from various substrata within the caves worldwide. These sequences correspond to a total of 445 fungal species, members of the 151 genera. Countries with the highest numbers of NCBI entries belonging to representatives of cave mycobiota, determined through geo_loc_name (qualifier of geographic location of the collected sample), are Spain, China, the USA, and France, followed by Brazil, Poland, and Slovakia (Figure 1).

### 3.1. Taxonomy of Cave Mycobiota—Dominant Phyla

**Figure 2:**
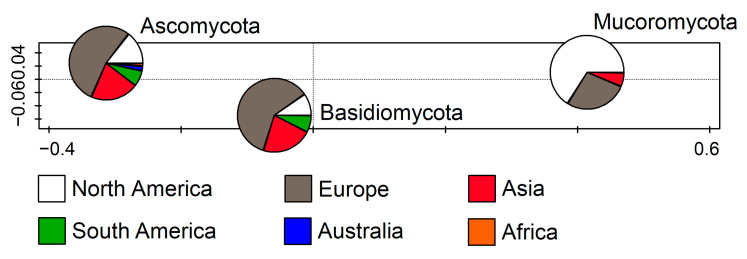
Redundancy analysis (RDA) showing fungal phyla from cave environments in relation to the continent.

The retrieved sequences represent three fungal phyla: Ascomycota, Basidiomycota, and Mucoromycota. Up to 1074 sequences correspond to Ascomycota (83.45%), while Mucoromycota (10.33%) and Basidiomycota members (6.22%) were encountered far less frequently. According to the RDA (F = 28.7, *p* = 0.002), which represents the relations between the fungal phyla and the continent on which they were recorded, Ascomycota and Basidiomycota were the most frequently recorded in Europe followed by Asia, while Mucoromycota were mostly documented in North America followed by Europe (Figure 2).

**Figure 3:**
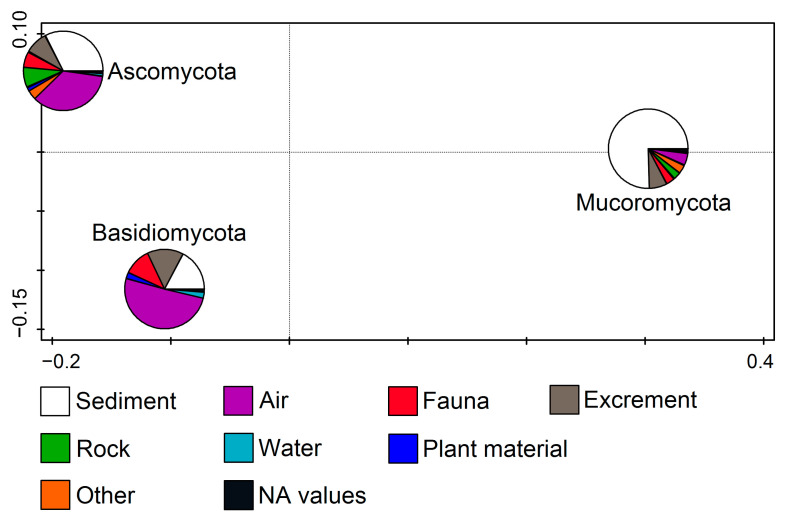
Redundancy analysis (RDA) showing fungal phyla from cave environments in relation to the substratum.

Fungal phyla in relation to the substratum (RDA, F = 11.1, *p* = 0.002) show that Ascomycota members were related to the air and sediment samples and the Basidiomycota mostly to the air, while the Mucoromycota isolates were mainly found in the sediment samples (Figure 3).

### 3.2. Dominant Classes of Fungi in Caves

**Figure 4:**
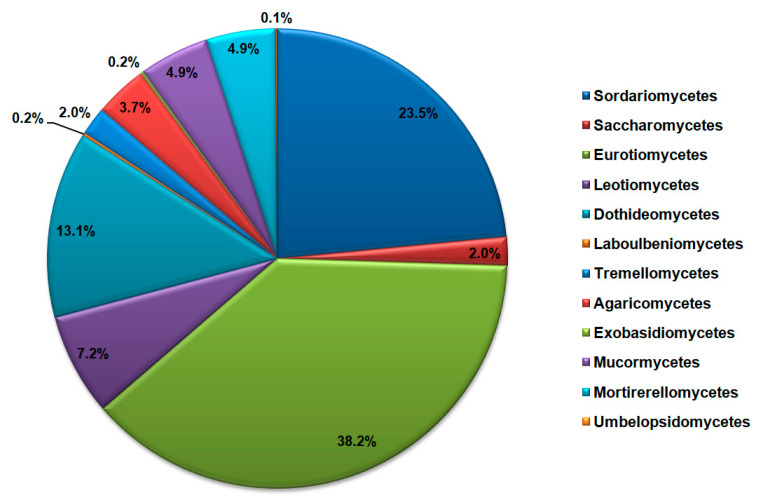
Most dominant classes of fungi in the caves according to the NCBI database search, represented as the total number of DNA sequences.

Phylum Ascomycota encompasses sequences of species belonging to classes Sordariomycetes (29.52% of all Ascomycota), Saccharomycetes (2.51%), Eurotiomycetes (47.88%), Leotiomycetes (9.03%), Dothideomycetes (16.48%), and Laboulbeniomycetes (0.19%). On the other hand, sequences of the Basidiomycota phylum were from classes Tremellomycetes (33.75% of all Basidiomycota), Agaricomycetes (62.50%), and Exobasidiomycetes (3.75%), while the phylum Mucoromycota encompasses sequences belonging to classes Mucormycetes (49.62% of all Mucoromycota), Mortirerellomycetes (49.62%) and Umbelopsidomycetes (0.01%). The most dominant classes of cave mycobiota according to the NCBI database sequences search are shown in Figure 4.

**Figure 5:**
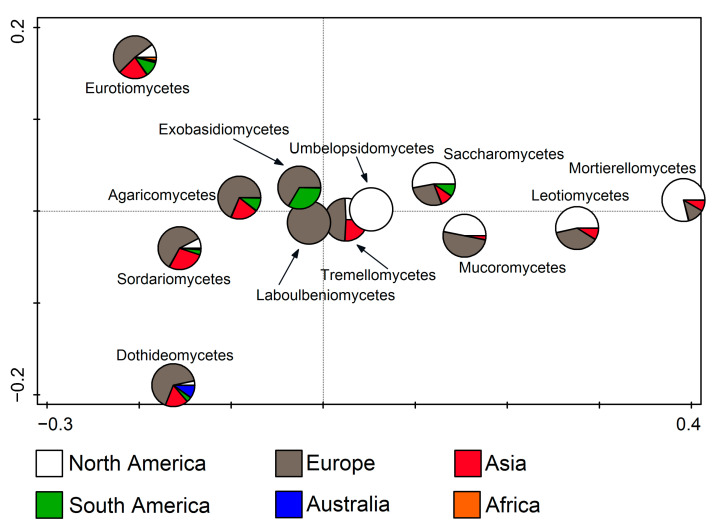
Redundancy analysis (RDA) showing fungal classes from cave environments in relation to the continent.

RDA was also used to relate fungal classes to the continents (F = 11.9, *p* = 0.002). It was noted that Laboulbeniomycetes members were found exclusively in Europe, and Umbelopsidomycetes in North America. Furthermore, it was clearly recognizable that Eurotiomycetes, Agaricomycetes, Sordariomycetes, Dothideomycetes, Tremellomycetes, Mucoromycetes, and Exobasidiomycetes entries dominate in Europe, while Saccharomycetes, Leotiomycetes, and Mortierellomycetes mostly occur in North America. In addition to these two continents, Asia stands out with Eurotiomycetes, Agaricomycetes, Sordariomycetes, Dothideomycetes, Tremellomycetes entries, and South America with Exobasidiomycetes. (Figure 5).

**Figure 6:**
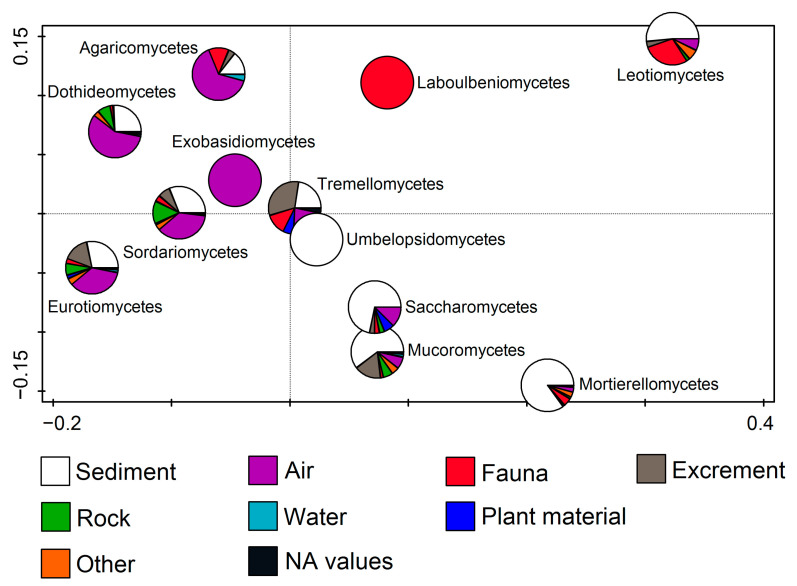
Redundancy analysis (RDA) showing fungal classes from cave environments in relation to the substratum.

By observing fungal classes in relation to the substratum, RDA (F = 7.8, *p* = 0.002) showed that members of some classes were connected to only one substratum, while others dominated on multiple substrata. Exobasidiomycetes were related only to air, while Agaricomycetes, Dothideomycetes, Sordariomycetes, and Eurotiomycetes were mostly related to air, followed by the sediment. Members of Umbelopsidomycetes were only found on sediment. Saccharomycetes, Mucoromycetes, and Mortierellomycetes members dominated on sediments, while Tremellomycetes were mostly related to sediments and excrements (including guano). Finally, Leotiomycetes dominated on sediment, followed by fauna (including many bats), while Laboulbeniomycetes representatives were only found on fauna (Figure 6).

### 3.3. Dominant Fungal Genera in Caves

**Figure 7:**
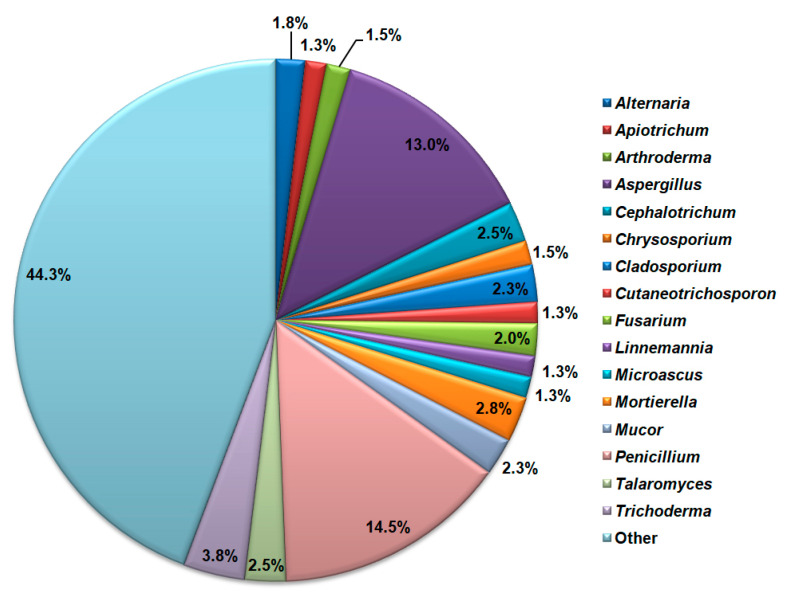
Cave mycobiota genera with the highest species richness according to the NCBI database search.

All NCBI-retrieved sequences belong to 151 fungal genera. The highest species richness is detected for the genus Penicillium with 57 detected species, followed by the genus Aspergillus with 51 detected species of cave mycobiota (Figure 7). *Trichoderma*, *Mortierella*, *Talaromyces*, *Mucor*, and *Fusarium* genera also had a significant number of cave mycobiota species.

### 3.4. Dominant Fungal Species in Caves

**Figure 8:**
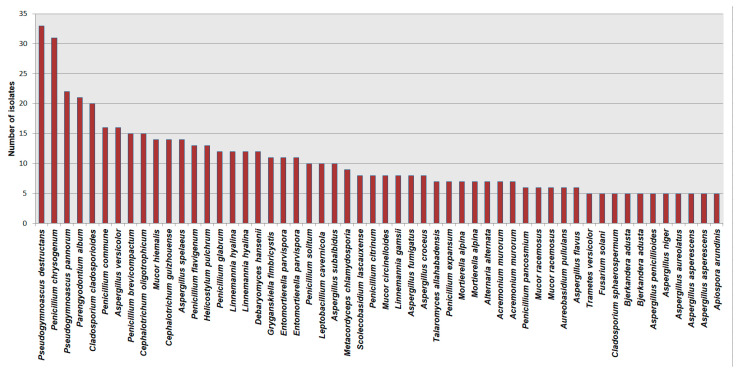
The list of dominant cave mycobiota species according to the NCBI database.

The most frequently encountered species is *Pseudogymnoascus destructans*, isolated mostly from hibernating bats and guano (33 hits), followed by Penicillium chrysogenum (31 hits). The list of the most dominant cave mycobiota species worldwide is presented in Figure 8.

**Figure 9:**
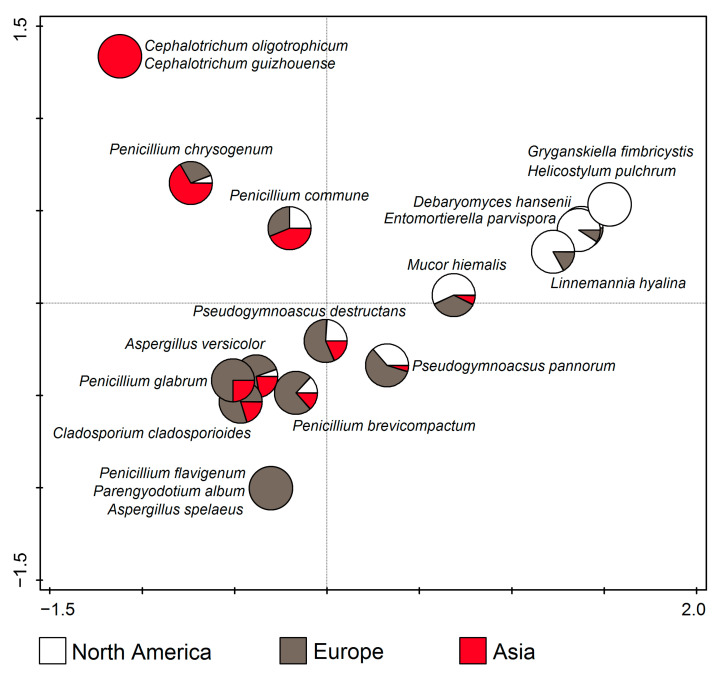
Canonical correspondence analysis (CCA) showing dominant fungal species from cave environments in relation to the continent.

According to RDA (F = 10.0, *p* = 0.002), *Cephalotrichum oligotrophicum* and *C. guizhouense* were only found in Asia, *Aspergillus spelaeus*, *Parengyodotium album*, and *Penicillium flavigenum* in Europe, and *Gryganskiella fimbricystis* and *Helicostylum pulchrum* in North America. Apart from that, the taxa in the upper left part of the ordination diagram dominated in Asia, those in the upper right in North America, while those in the lower half of the diagram dominated in Europe. (Figure 9).

### 3.5. New Fungal Species Described in Caves

The NCBI database search revealed the presence of 43 fungal species detected for the first time in caves (Table 1). The genus with the most described cave species was *Aspergillius*, with seven species first reported from various cave substrata (cave, air, sediment, guano) in Spain, Romania, Brazil, China, and Botswana. Also, cave species richness was documented for the genera *Apiotrichum* and *Cephalotrichum*, with both having four species described for the first time in cave environments. All newly discovered *Apiotrichum* species are from bat guano in Akiyoshi-do cave in Japan. On the other hand, three new species of genus *Cephalotrichum* were recorded for stone substratum in China, while the *C. lignatile* is isolated from timber in a cave in Belgium. The substrata with the most reported new fungal species are guano and stone.

## 4. Discussion

This study represents the first attempt to create a review regarding cave mycobiota via NCBI data search accompanied by a statistical approach. These types of papers, with catalogs of DNA sequences, serve as comprehensive compilations of genetic information about a group of organisms that are of interest. In fact, the significance of these studies lies in the fact that they organize data from various manuscripts and databases, making it easier for researchers to access, analyze, and utilize the information. Additionally, since it is very well known that NCBI and other biotechnology repositories contain vast amounts of genomic data, review papers that catalog this information provide a centralized resource, making it more accessible even for those researchers who may not have the time or expertise to navigate complex databases. It should be noted that analyzing a large set of sequences collectively can reveal insights that may not be apparent when examining individual sequences in a sole study. Finally, reviews of this type provide a resource for those working on computational analysis, allowing them to test algorithms, develop models, and draw conclusions based on a well-curated set of genetic data.

The majority of NCBI entries of cave mycobiota are sequences belonging to the members of Ascomycota (83.45%). This is an expected result since this is the fungal phylum with the highest species richness, dominating in both described and estimated global fungal diversity. It contains over 64,000 described species, which is more than 75% of all known fungi [108]. The actual number is likely much higher, with estimates exceeding 2 million species [109]. Namely, the isolation of fungi from various substrata, especially in extreme environments, such as caves, deserts, deep sea habitats, and even Antarctic soils, always results in a high diversity of Ascomycota compared to other fungal phyla [110,111,112]. Unlike Basidiomycota taxa, which need substrata rich in nutrients (such as wood and dung) and often rely on symbiotic relationships with plants, or are plant pathogens, ascomycetes do not depend on photosynthetic organisms and can thrive in complete darkness [48]. Furthermore, many ascomycetes produce antifungal or antibacterial compounds that help them outcompete other microorganisms, giving them a selective advantage in caves where microbial competition is high [113]. Also, many ascomycetes can form SAB [114] and live as endolithic or epilithic fungi [115], contributing to the microbial communities on various stone substrata within the caves, such as cave walls, stalagmites, and stalactites. Due to all these traits (protective secondary metabolites and ability to form SAB), ascomycetes can not only outcompete Basidiomycota members but can also outcompete species of Mucoromycota, which are less competitive in oligotrophic conditions.

According to the RDA analyses, members of the phylum Mucoromycota are predominantly documented in North America. On the other hand, *Histoplasma capsulatum*, the causative agent of Darling’s disease, is also often documented in Central and Eastern United States caves with high organic matter, particularly in bat and bird guano deposits [116]. It appears that Mucoromycota members are often documented in North American caves where *H. capsulatum* is reported, and while both thrive in caves with high nitrogen organic content matter, such as guano deposits [117], benefiting from similar environmental conditions; their ecological roles and frequencies differ [118]. It should also be noted that in studies of cave mycobiota conducted in North America, *Mucoromycota* species could be detected due to sampling bias in caves with potential risks of histoplasmosis outbursts.

RDA analyses additionally showed that propagules of Ascomycota and Basidiomycota (conidia or basidiospores) are more frequently encountered in cave air than the sporangiospores of Mucoromycota. Members of Ascomycota and Basidiomycota phyla actively discharge their spores into the air either by producing dry, hydrophobic conidia that easily become airborne or by ballistic discharge [119]. On the other hand, sporangiospores of Mucoromycota species are often passively released when sporangia ruptures, which often requires mechanical disturbance (e.g., wind, animal movement, or water flow). Furthermore, Mucoromycota species sporangiospores tend to be sticky, larger, and settle quickly, reducing their presence in airborne cave samples [120]. On the contrary, many spores of Ascomycota and Basidiomycota are smaller (~2–10 µm), lightweight, and can remain suspended in the air for long periods of time [121], unlike Mucoromycota sporangiospores, which are larger (often >10 µm), heavier, settle more quickly, tend to aggregate, and are less likely to be widely dispersed in still cave air. This in mind, Mucoromycota spores tend to stay near their source (e.g., soil, guano, decaying material) unless disturbed by human activity or animal movement [122]. Also, many cave mycological studies use airborne spore sampling with sedimentation plates or air samplers, which favor fungi that release lightweight spores.

According to the NCBI database search, the most dominant fungal classes within the caves are Eurotiomycetes and Sordariomycetes. This is mostly due to their adaptations to oligotrophic and extreme environments. The most frequently encountered genera in caves belonging to the class Eurotiomycetes are *Aspergillus* and *Penicillium*, which are characterized by thick-walled spores (conidia) that can survive harsh conditions, including desiccation and low nutrient availability [122]. Some Eurotiomycetes are commonly found in soil, dust, and on animals, allowing them to be introduced into caves through human visitation or animal migration [123]. On the other hand, many Sordariomycetes, such as *Fusarium*, *Trichoderma*, and *Chaetomium*, are decomposers that break down organic matter like guano, plant debris [124], or even keratinous material from animal remains [125]. Therefore, their ability to degrade complex organic compounds allows them to persist in caves where nutrient sources are scarce. Similarly, Sordariomycetes produce resistant spores (ascospores and conidia) that can remain dormant for long periods and germinate when conditions become favorable. Air currents, water, animals, and even human visitors can introduce and disperse these spores. It is known that bat and bird guano often serve as hotspots for fungal activity, including many Sordariomycetes. Species like *Fusarium* and *Beauveria* have been found in guano deposits, where they contribute to nutrient cycling and sometimes even act as opportunistic pathogens of insects [48,126]. According to RDA analyses, only two fungal classes are linked with specific substratum. Namely, Laboulbeniomycetes were recorded only on fauna elements in Europe, while Exobasidiomycetes were exclusively airborne. The NCBI database search revealed the presence of only two entries of Laboulbeniomycetes in caves, both members of the genus *Arthrorhynchus* (*A. eucampsipodae* MT241715 and *A. nycteribiae* MT235715) recorded on bat flies *Nycteribia vexata* and *Penicillidia conspicua* parasitizing chiropterans *Myotis blythii* and *M. schreibersii* in Mandrata Cave in Bulgaria [88]. Representatives of the class Exobasidiomycetes documented in this study (members of genera *Sympodiomycopsis*, *Tilletiopsis*, and *Golubevia*) are exclusively associated with plants, as saprobes or pathogens—causative agents of galls (abnormal outgrowths of plant tissues) [127]—and as such cannot colonize any substratum within the caves. Therefore, these species could be regarded as trogloxenic. Furthermore, these fungi produce a variety of fungal spores, among them basidiospores and balistospores, which could be dispersed by air currents and as such detected via air sampling devices. Finally, there is only one NCBI entry of Umbelopsidomycetes, exclusively found in the cave of North America (*Umbelopsis isabellina* KC009121) from bat sediments [61].

Genera *Penicillium* and *Aspergillus* showed the highest species richness in caves worldwide. Members of both genera can break down a wide range of organic materials, including guano, plant debris, and even mineral substrata. Some species can utilize nitrogen sources from bat guano effectively and were found to be dominant in this substratum [128]. It is well known that *Aspergilli* and *Penicillia* produce airborne conidia that are highly resistant and easily dispersed by air currents, water, and animal movements, allowing them to colonize different cave microhabitats. Many *Penicillium* species can tolerate low temperatures, high humidity, and nutrient-poor environments, which are common in caves. Some also exhibit resistance to desiccation. Conversely, *Aspergillus* species, particularly xerophilic ones (e.g., *A. niger*, *A. flavus*), are well adapted to extreme cave conditions, including low water availability and oligotrophic environments [122]. Species belonging to both genera produce antifungal, antibacterial, and mycotoxic compounds, which allow them to compete with other cave microorganisms [122,129]. Furthermore, both genera benefit from the relatively stable temperature and humidity levels found in caves, which support their slow but steady growth. While *Penicillium* species tend to dominate in cooler and more humid cave environments, *Aspergillus* species (especially *A. niger*, *A. flavus*, and *A. fumigatus*) are more frequently found in drier, warmer conditions and are more tolerant to low water activity [130]. Some *Aspergillus* species such as *A. fumigatus* (eight NCBI entries in research presented here) are opportunistic pathogens that can affect birds and humans, especially in enclosed cave environments with high spore concentrations [11,61]. On the contrary, *Penicillium* species generally pose less of a health risk, although their spores can be allergenic [131]. The *Penicillium* species with the most cave mycobiota NCBI hits is *P*. *chrysogenum*. The most entries in the NCBI database of this species are from China and Spain, and their sources are, in the majority of cases, sediment, air, and rock substratum. The presence of this species on rock substratum must not be neglected since this species has the potential to deteriorate limestone substrata, including cave walls [132,133].

*Pseudogymnoascus destructans* is the most frequently encountered fungal species documented during the NCBI database search with 33 hits. Most of the entries are from the USA, Italy, and Russia, and bat species *Myotis lucifugus*, *Myotis emarginatus*, *Perimyotis subflavus*, and *Myotis dasycneme* are listed as hosts. The majority of outbreaks of *P. destructans* occur primarily in temperate regions of North America and Europe, where cave-dwelling bats hibernate [134]. Detected for the first time in a cave in Albany (New York) in 2006, the fungus has since spread to over 35 US states and 7 Canadian provinces [135]. Although detected in various countries in Central and Eastern Europe, European bats seem to have coevolved with *P. destructans*, experiencing fewer mortality events compared to those inhabiting North America [136]. Although *P. destructans* is commonly found in caves and mines, where it infects hibernating bats, it has also been detected in non-cave environments, such as soil and bat roosts outside caves, and as such can be regarded as troglophilic or trogloxenic.

Many novel fungal species were documented in caves because these environments harbor low-competition, nutrient-limited, and stable microhabitats that favor unique or cryptic species. Additionally, improved sampling techniques and state-of-the-art molecular tools are uncovering previously unknown fungal diversity in caves around the world [14,104]. Additionally, caves contain microhabitats that are rarely sampled in surface ecosystems, increasing the likelihood of finding new species, e.g., bat guano piles, which are rich in nitrogen (therefore “attracting” unique fungal communities) [116]. However, it should be emphasized that some fungi isolated from caves may be cryptic species—closely related to known taxa but genetically distinct. Likewise, caves can act as refugia, preserving ancient fungal lineages that are extinct or rare in surface habitats [137]. Modern culture-independent techniques (e.g., metabarcoding, environmental DNA studies) allow researchers to detect fungi that are difficult to cultivate in laboratory conditions [138]. In fact, many previously undescribed fungal species are detected only after the molecular analysis of cave samples [14,24]. Finally, novel fungal species are often isolated because researchers are specifically looking for organisms with biotechnological potential (e.g., antibiotic production, the biodegradation of pollutants) [15,20].

To overcome the restrictions of the low availability of nutrients in cave ecosystems, microbial populations create collective structures (such as various kinds of biofilms) where they collaborate and establish mutualistic connections rather than fighting for resources. This is the reason why the majority of environmental microorganisms (including fungi) cannot grow in laboratory settings due to the fact that their growth is dependent on certain interactions with other species and cannot occur on its own [139]. Furthermore, fungi and other microorganisms in caves often rely on alternative metabolic pathways, such as breaking down unusual organic compounds [140]. Many psychrotolerant and xerotolerant fungi thrive in caves, including species that may go unnoticed in drier surface environments [110]. Some cave fungi exhibit adaptations similar to extremophiles, tolerating high salinity, heavy metals, or radiation [141]. Therefore, it could be stated that caves act as “natural laboratories for extremophiles”.

## Figures and Tables

**Table 1:** Novel fungal species isolated from various cave substrata according to NCBI search.

Species	Accession Numbers	Country	Cave	Substratum	Ref.	Holotype
*Amphichorda cavernicola*	MK336089 MK336003	China	Feng Cave, Sichuan, Xingwen	Bird feces	[104]	HMAS 24801 (From dung of Aves in cave: Sichuan)
*Apiotrichum akiyoshidainum*	AB180200	Japan	Akiyoshi-do	Guano	[105]	JCM 12595
*Apiotrichum chiropterorum*	AB180197	Japan	Akiyoshi-do	Guano	[105]	JCM 12594
*Apiotrichum coprophilum*	AB180199	Japan	Akiyoshi-do	Guano	[105]	JCM 12596
*Apiotrichum otae*	AB180196	Japan	Nippara-shonyud	Guano	[105]	JCM 12593
*Aspergillus cavernicola*	HG008744	Romania	Caprei grotto	Cave walls	[91,107]	-
*Aspergillus croceus*	LN873932, LN873931	Spain	Cueva del Tesoro	Sediment	[73]	PRM 924053 (From cave sediment: Spain)
*Aspergillus dobrogensis*	LT626976	Romania	Movile Cave	Sediment	[99]	PRM 935751 (From cave sediment: Romania)
*Aspergillus lebretii*	ON862927 ON862928	Brazil	The Abrigo do Letreiro Cave	Air	[35]	URM 95150 (From air, in cave: Rio Grande do Norte)
*Aspergillus limoniformis*	NG_075262	China	Mingjiu Cave, Yunang	Bat guano	[104]	HMAS 248014 (From dung of Chiroptera in cave: Yunnan)
*Aspergillus okavangoensis*	MW480788	Botswana	Gcwihaba Caves, Okavango basin	Bat guano-contaminated soil	[74]	PREM 63212 (From soil contaminated with feces of Chiroptera: Botswana)
*Aspergillus phialiformis*	MK336096	China	Sanjiao Cave, Yunan	Stone	[104]	HMAS 248017 (From rocks in cave: Yunnan)
*Blastobotrys persicus*	NG_088046	Iran	Mejare Cave	Soil	[93]	IBRC-M 30238 (Isolated from cave soil: Iran)
*Cephalotrichum guizhouense*	MF419783 MF419752	China	Cave in Shuanghe National Geographic Park, Guizhou Province	Stone	[83]	HMAS 247177 (From limestone from cave: Guizhou)
*Cephalotrichum laeve*	MF419780 MF419750	China	Cave in Shuanghe National Geographic Park, Guizhou Province	Stone	[83]	HMAS 247178 (From limestone from cave: Guizhou)
*Cephalotrichum lignatile*	KY249269 NR_154842	Belgium	-	Timber	[25]	CBS H-22852: (Isolated from timber in cave: Belgium)
*Cephalotrichum oligotrophicum*	NG_069510	China	Cave in Shuanghe National Geographic Park, Guizhou Province	Stone	[83]	HMAS 247176 (From limestone from cave: Guizhou)
*Chrysosporium speluncarum*	AM949569	Slovakia	Jasovska Cave	Guano heap of insectivore bat *Rhinolopus euryale*	[75]	-
*Cutaneotrichosporon cavernicola*	AB180195	Japan	Nippara-shonyud	Bat guano	[105]	JCM 12590
*Cutaneotrichosporon middelhovenii*	NR_172214 AB180198	Japan	Nippara-shonyud	Bat guano	[105]	JCM 12592
*Debaryomyces psychrosporus*	HM769277 NG_066358 NG_064957 HM769276 HM769275	Venezuela	Crystal Eyes Cave (Cueva Ojos de Cristal), Roraima Tepui Mountain	Secondary mineral deposits (stalactites and stromatolites)—silicates	[33]	NCAIM Y.01972 (Isolated from dry stalactites of silicic sandstone: Venezuela)
*Endophoma elongata*	NG_070363 JF340088	Canada	Cadomin Cave	Bat cave soil	[82]	UAMH 11216 (Isolated from soil of bat cave: Alberta)
*Gymnascella minnisii*	MW054470 MW054469 MT988379	USA	Canoe Creek Hartman Mine, Pennsylvania, Blair County	Bat guano	[49]	CUP 70725
*Gymnoascus exasperatus*	NR_178112 NG_088047 KY883236	China	-	Bat guano	[14]	HMAS 246925 (Isolated from guano of bat: Guizhou)
*Leptobacillium cavernicola*	OM622527 OM628847 OM628786	France	Paleolithic-decorated cave (Pair-non-Pair cave)	Cave wall	[44]	CBS 149113 (From rock, in cave: France)
*Malbranchea cavernosa*	ON862930 ON862923	Brazil	The Abrigo do Letreiro	Air	[35]	URM 95151 (From air, in cave: Rio Grande do Norte)
*Microascus globulosus*	KY883283 KY883243 KX855234	China	Unnamed karst Cave	Guano	[14]	HMAS 246928 (Isolated from bat guano: Guizhou)
*Mortierella multispora*	MT031921 NG_075335 MT032146	China	Cave outside of Kunming City, Yunnan Province	Carcass of *Rhinolophus affinis* (bat)	[62]	YMF 1.06174
*Mortierella rhinolophicola*	MT031919 NG_075334 MT032144 NR_172422	China	Cave outside of Kunming City, Yunnan Province	Carcass of *Rhinolophus affinis* (bat)	[64]	YMF 1.06175
*Mortierella yunnanensis*	MT031917 NG_075333 MT032142 NR_172421	China	Cave outside of Kunming City, Yunnan Province	Carcass of *Rhinolophus affinis* (bat)	[64]	YMF 1.06176
*Neocosmospora pallidimors*	MT031916 MT032141	China	Cave outside of Kunming City, Yunnan Province	Carcass of *Rhinolophus affinis* (bat)	[64]	YMF 1.06177
*Neopestalotiopsis cavernicola*	MW581238 MW545802	China	Gem Cave, inner cave	Rock surfaces	[39]	HKAS 111937 (From surface of rocks, in cave: Yunnan)
*Paraboeremia oligotrophica*	KX829041 KX829040 KX829039	China	Cave at the Shuanghe National Geographic Park, Guizhou Province	Limestone	[89]	HMAS 247036 (Isolated from carbonatites from cave: Guizhou)
*Penicillium speluncae*	MG490867	Canada	White Cave	Cave wall	[24]	DAOM 745788
*Penicillium vanluykii*	JX997007	USA	Lechuguilla Cave	-	[28]	CBS H-21059 (Isolated from cave: New Mexico)
*Pseudogymnoascus palmeri*	MT988150	USA	Woodward Cave	Sediment	[49]	CUP-70724
*Pseudolecanicillium caatingaense*	ON862934 ON862926	Brazil	The Abrigo do Letreiro Cave	Air	[35]	URM 95152 (From air, in cave: Rio Grande do Norte)
*Scolecobasidium anellii*	FR832477 NR_111436	Italy	Castellana Cave	Superficially darkened stalactites	[81]	-
*Scolecobasidium anomalum*	HE575202	France	Lascaux Cave, Paleolithic paintings	Black stain on cave sediment	[81]	-
*Scolecobasidium lascauxense*	HE575200	France	Lascaux Cave, Paleolithic paintings	Black stain on cave sediment	[81]	-
*Setophoma caverna*	MK511944 MK511965	China	-	Rock	[50]	HMAS 248085
*Simplicillium pechmerlense*	MW031272 MW031740 MW031268	France	Pech-Merle Cave	Air	[68]	CBS 147188 (From air in cave: France)
*Talaromyces cavernicola*	ON862936 ON862935	Brazil	The Abrigo do Letreiro Cave	Air	[35]	URM 95155 From air, in cave: Rio Grande do Norte

## Data Availability

No new data were created or analyzed in this study.

## Supplementary Materials

The following supporting information can be downloaded at https://www.mdpi.com/article/10.3390/jof11040286/s1: Supplementary Table S1 (List of NCBI entries of fungi from cave environments); Supplementary File S1.
