# Supplementary material for: Unveiling the Subterranean Symphony: A Comprehensive Study of Cave Fungi Revealed Through National Center for Biotechnology Sequences
Source: J Fungi (Basel). 2025 Apr 5;11(4):286. doi: 10.3390/jof11040286 (PMC12028181; doi:10.3390/jof11040286)
Supplement: Supplementary file 1 [file jof-11-00286-s001.zip › Supplementary File S1.pdf]

**Sediment** (Cave sediment, cave soil, Mn rich sediment, soil and sediment, soil sediment containing of powdered basalt and guano, sediment, sediments, sediment with sings of bat guano, soil, soil and sludge, sediment rich in bat guano, sediment keratin bait, bat guano contaminated soil, differently colored stains on save sediment, bat cave soil);

**Rock** (Cave wall, cave rock, rock wall, secondary mineral deposits (stalactites, stalagmites), inner cave rock surfaces, weathered rocks, carbonatite, superficial darkened stalactite, limestone, bat cave wall, paintings, stone);

**Excrement** ((Guano) Bat guano, bat dong, vampire bat guano (Other excrement Porcupine, rodent, mammal, invertebrate, diplopod, earthworm casts);

**Fauna** ((Bat), superficial sampling from wings, ears, muzzles (Other fauna) Moth, fly, millipede;

**Air** (air inside, cave air, air, airborne);

**Plant material** ((Plant litter) Leaf litter, plant debris (Wood) Timber)

**Water** (Cave watre, water);

**Other** (rock/water, air/rock, sediment/rock, air/rick/water, air/water, painting/sediment, snottite, spider web with algae *Dunaliella*, dust with bat guano, surface sampling, Art gallery, environmental swab, flooe (Bones) Bones of paleolithic bear).
